# Anthracycline Therapy Modifies Immune Checkpoint Signaling in the Heart

**DOI:** 10.3390/ijms24076052

**Published:** 2023-03-23

**Authors:** Sebastian Korste, Stephan Settelmeier, Lars Michel, Andrea Odersky, Pia Stock, Fabrice Reyes, Elias Haj-Yehia, Markus S. Anker, Anika Grüneboom, Ulrike B. Hendgen-Cotta, Tienush Rassaf, Matthias Totzeck

**Affiliations:** 1 Department of Cardiology and Vascular Medicine, West German Heart and Vascular Center, University Hospital Essen, 45147 Essen, Germany; 2 Deutsches Herzzentrum der Charité, Department of Cardiology, Angiology and Intensive Care Medicine, 12203 Berlin, Germany; 3 Charité—Universitätsmedizin Berlin, Corporate Member of Freie Universität Berlin and Humboldt-Universität zu Berlin, 10117 Berlin, Germany; 4 Berlin Institute of Health Center for Regenerative Therapies (BCRT), 13353 Berlin, Germany; 5 German Centre for Cardiovascular Research (DZHK), Partner Site Berlin, 10785 Berlin, Germany; 6 Leibniz-Institut für Analytische Wissenschaften—ISAS—e.V., 44139 Dortmund, Germany

**Keywords:** cardio-oncology, cardiotoxicity, doxorubicin, immune checkpoint inhibitor, programmed cell death ligand 1

## Abstract

Cancer survival rates have increased significantly because of improvements in therapy regimes and novel immunomodulatory drugs. Recently, combination therapies of anthracyclines and immune checkpoint inhibitors (ICIs) have been proposed to maximize neoplastic cell removal. However, it has been speculated that a priori anthracycline exposure may prone the heart vulnerable to increased toxicity from subsequent ICI therapy, such as an anti-programmed cell death protein 1 (PD1) inhibitor. Here, we used a high-dose anthracycline mouse model to characterize the role of the PD1 immune checkpoint signaling pathway in cardiac tissue using flow cytometry and immunostaining. Anthracycline treatment led to decreased heart function, increased concentration of markers of cell death after six days and a change in heart cell population composition with fewer cardiomyocytes. At the same time point, the number of PD1 ligand (PDL1)-positive immune cells and endothelial cells in the heart decreased significantly. The results suggest that PD1/PDL1 signaling is affected after anthracycline treatment, which may contribute to an increased susceptibility to immune-related adverse events of subsequent anti-PD1/PDL1 cancer therapy.

## 1. Introduction

New antineoplastic therapies have dramatically improved the long-term survival rates of cancer patients in recent years [1]. Conventional chemotherapeutics target rapidly dividing cells via the inhibition of cell division, e.g., by intercalation to DNA. In contrast, targeted and immune therapies affect inter- and intracellular signaling pathways and immune cells, resulting in lower tumor burden [2]. In addition to higher survival rates, many adverse effects of these therapies have been found, of which cardiotoxic side effects are among the most lethal in this group [3,4]. 

Conventional anthracycline cancer therapeutics, e.g., Doxorubicin (DOX), belong to a family of antibiotics used to treat solid tumors and hematologic malignancies. They are well known for their acute and chronic cardiotoxic side effects that finally result in cardiotoxic cardiomyopathy and congestive heart failure, potentially limiting their clinical use [5,6]. Mechanisms of anthracycline cardiotoxicity are considered multifactorial, and the global understanding of the underlying mechanisms is still incomplete. Pathomechanisms include the binding of topoisomerase IIβ in cardiomyocytes, the generation of reactive oxygen species due to direct and indirect stress damage to cardiomyocyte mitochondria, impaired ion handling, and disrupted mitochondrial quality control [4,7,8]. In some cases, anthracyclines are applied in combination regimens with novel cancer therapies including ICI. Interestingly, anthracyclines have been found to affect immune checkpoint signaling pathways targeted by these subsequently applied therapeutics [9,10,11,12,13].

The integration of inhibiting and activating signals at the level of T cells determines the effectivity of the cellular immune response against tumor cells [14]. T cells recognize tumor-specific antigens via their T cell receptors, initiating their anti-tumor cell activity [15]. PD1, an inhibitory checkpoint molecule, is responsible for limiting a permanently activated T cell response, thus protecting healthy as well as tumor cells from an overshooting immune response. ICI against PD1 belongs to the family of immune therapies and promotes a distinct lasting antitumor activity in T cells in solid tumors and hematologic malignancies via releasing the brake of the immune system [16,17]. However, ICI therapy inherits the risk of immune-related adverse events, of which myocarditis is the most severe therapy-limiting or in many cases fatal form [16,18,19]. Recently, PDL1 on cardiac endothelial cells has been identified as the main mediator of immune crosstalk in a mouse model of anti-PD1 therapy, which promoted myocardial infiltration with inflammatory cells preceding apparent cardiotoxicity [20].

Synergistic therapeutic regimens are used in the treatment of advanced cancer. Here, the sequential combination of conventional and immune therapies in advanced cancer patients can improve patient outcomes, e.g., as a treatment for triple-negative breast cancer [21,22,23,24,25,26]. Due to the still infrequent but expected increasing use of combination therapies, information on clinical outcomes is limited. Nevertheless, PD1-PDL1 interaction plays a crucial role in maintaining cardiac immunological integrity and is involved in various forms of cardiac injury. However, its role in patients pre-treated with anthracycline has not yet been characterized.

The amplifying effect of the combination therapy in tumor tissue may trigger increased susceptibility for ICI-related cardiotoxicity, further increasing anthracycline-induced cardiotoxicity with considerable consequences on cardiovascular morbidity and mortality. Therefore, this study aims to elucidate the response of PD1/PDL1 signaling to anthracyclines exposure.

## 2. Results

### 2.1. DOX Treated Mice Show a Profound Effect on Cardiac Function

**Figure 1:**
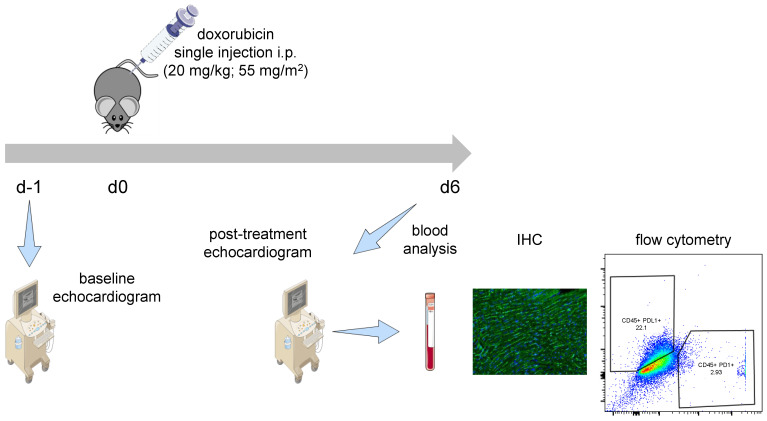
Treatment regime. After initial transthoracic echocardiography, mice received a single intraperitoneal (i.p.) injection of Doxorubicin (20 mg/kg; 55 mg/m^2^). After 6 days, heart function was re-evaluated using echocardiography. Mice were sacrificed and the effects of Doxorubicin on cardiac PD1/PDL1 signaling were analyzed using flow cytometry and immunohistochemistry.

To study the effects of anthracycline therapy on the PD1/PDL1 pathway, mice were injected with a single high-dose of Doxorubicin (20 mg/kg; 55 mg/m^2^) after baseline echocardiographic assessment. After 6 days, heart function was measured and cardiac PD1/PDL1 signaling was evaluated (Figure 1) and compared to baseline.

**Figure 2:**
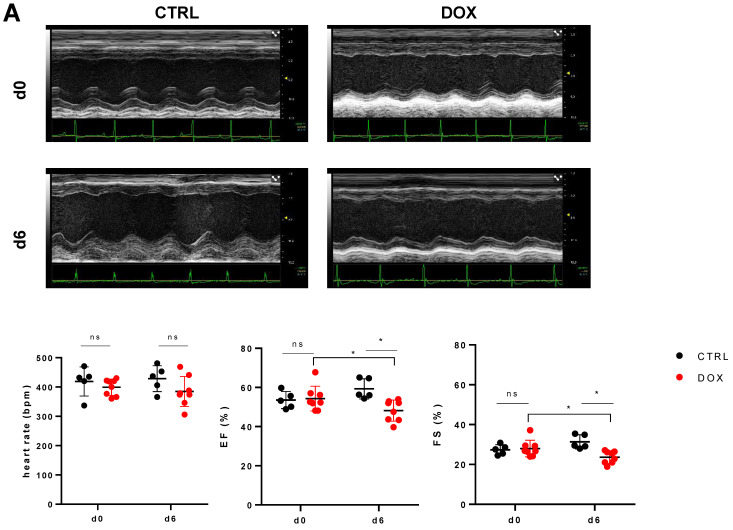

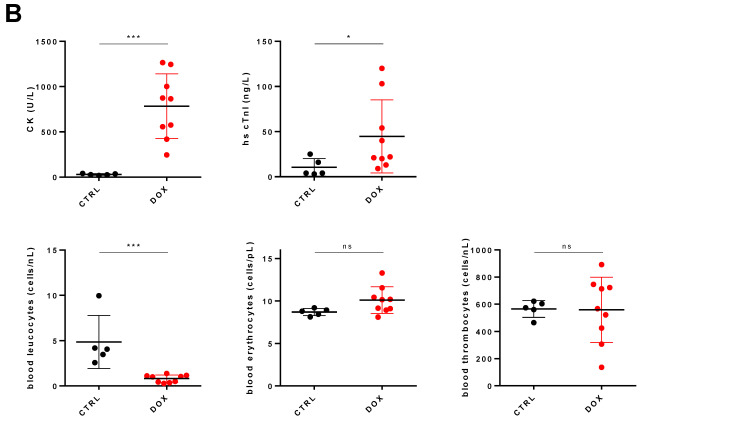
Effects of Doxorubicin treatment on heart function and blood parameters. (**A**) Top Exemplary M-mode echocardiograms from parasternal short axis views of the mouse heart of both the control (CTRL) and Doxorubicin (DOX) group before (d0) and after treatment (d6). Bottom Comparison of heart rate in isoflurane narcosis, as well as left ventricular ejection fraction (EF) and fractional shortening (FS) of CTRL and DOX treated animals (n = 5–8, ANOVA with Bonferroni post-hoc test, *: *p* < 0.05). (**B**) Top Levels of creatine kinase (CK) and highly sensitive cardiac troponin I (hscTnI) from control and doxorubicin treated animals at day 6 after beginning of experiment (n = 5–9, Student’s *t*-test with Welch correction, *: *p* < 0.05, ***: *p* < 0.001). Bottom Number of leucocytes, erythrocytes and thrombocytes present in the blood of CTRL and DOX groups at day 6 after injection (n = 5–9, Mann–Whitney test, ***: *p* < 0.001). ns: not significant.

The mice investigated six days after DOX treatment showed no change in heart rate during echocardiographic assessment compared to control mice (384.63 ± 47.75 bpm vs. 428.6 ± 39.6 bpm, *p* = 0.178, Figure 2A); furthermore, DOX-treated mice did not differ in heart rate from day 0 to day 6 (384.63 ± 47.75 bpm vs. 399.25 ± 26.04 bpm, *p* = 0.689, Figure 2A). However, heart function was reduced in the DOX-treated group compared to baseline in terms of both ejection fraction (EF: 48.16 ± 5.03% vs. 54.28 ± 5.92%, *p* = 0.036, Figure 2A) and fractional shortening (FS: 23.65 ± 2.9% vs. 27.98 ± 3.93%, *p* = 0.021, Figure 2A), as well as in comparison to the control group (EF: 48.16 ± 5.03% vs. 59.29 ± 4.62%, *p* = 0.004; FS: 23.65 ± 2.9% vs. 31.38 ± 3.15%, *p* = 0.002, Figure 2A). As a marker of myocardial injury, we found increased levels of creatine kinase (CK: 783.33 ± 337.38 U/L vs. 29.4 ± 7.79 U/L, *p* = 0.001, Figure 2B) and high-sensitive cardiac troponin I (hscTnI: 44.67 ± 38.22 ng/L vs. 10.4 ± 8.73 ng/L, *p* = 0.038, Figure 2B). Blood analysis revealed, as expected, decreased levels of circulating leucocytes (0.81 ± 0.39 cells/nL vs. 4.86 ± 2.61 cells/nL, *p* = 0.001, Figure 2B), while levels of erythrocytes and thrombocytes did not differ (erythrocytes: 10.10 ± 1.48 cells/pL vs. 8.70 ± 0.37 cells/pL, *p* = 0.065; thrombocytes: 559.11 ± 225.73 cells/nL vs. 565 ± 54.55 cells/nL, *p* = 0.937, Figure 2B).

### 2.2. PDL1 Expression following Anthracycline Treatment

**Figure 3:**
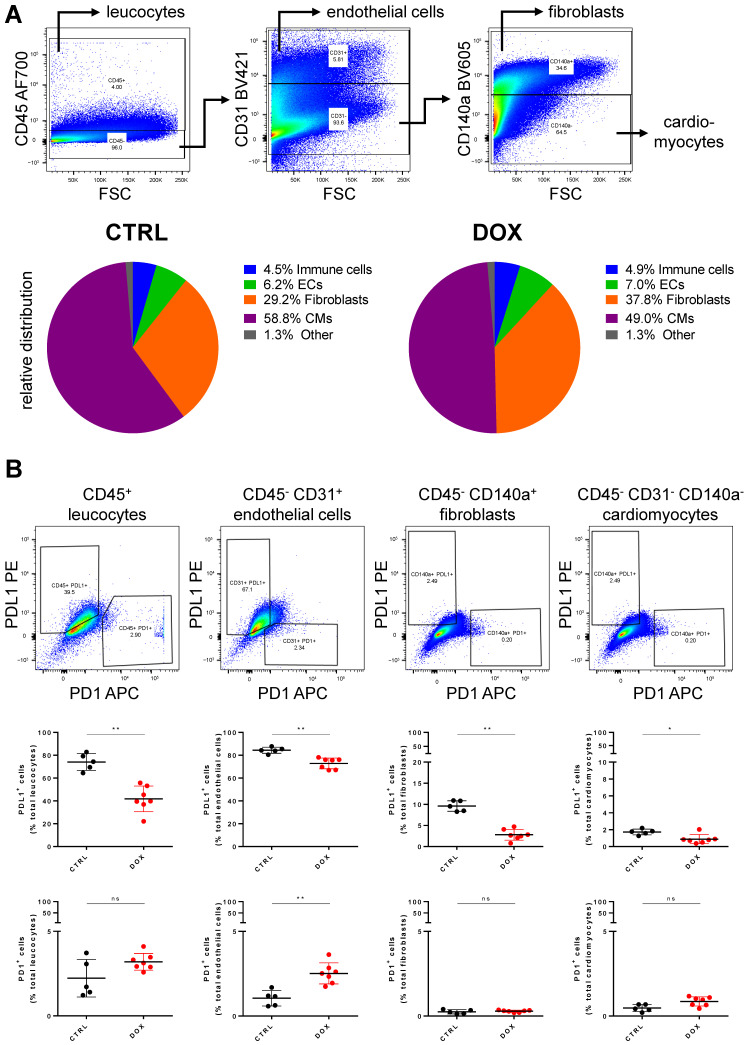
Doxorubicin modifies heart tissue composition and PDL1/PD1 expression on cardiac cells. (**A**) Top Exemplary pseudo-color dot plots for separation of cell populations in heart tissue using flow cytometry. Bottom Ratios of immune cells, endothelial cells (ECs), fibroblasts and cardiomyocytes (CMs) relative to total cell number measured in control (CTRL) and Doxorubicin (DOX) group. Differences in fibroblast and cardiomyocyte ratios is significant between both groups (n = 5–7, Student’s *t*-test, *p* < 0.05). (**B**) Top Exemplary pseudo-color dot plots for PD1/PDL1 expressing leucocytes, endothelial cells, fibroblasts and cardiomyocytes. Middle Percentage of PDL1 expressing cells of total leucocytes, endothelial cells, fibroblasts and cardiomyocytes from control (CTRL) and Doxorubicin (DOX) treated animals (n = 5–7, Mann–Whitney test, *: *p* < 0.05, **: *p* < 0.01). Bottom Percentage of PD1 expressing cells of total leucocytes, endothelial cells, fibroblasts and cardiomyocytes from control (CTRL) and Doxorubicin (DOX) treated animals (n = 5–7, Mann–Whitney test, **: *p* < 0.01). ns: not significant.

We next investigated cardiac tissue composition and the expression of PD1 and PDL1 on these different cell populations using flow cytometry. We found a decreased number of cardiomyocytes relative to the total number of cells in DOX-treated mice (49.03 ± 4.60% vs. 58.79 ± 6.66%, *p* = 0.021, Figure 3A). However, the number of immune cells and endothelial cells was consistent in proportion in both groups (immune cells: 4.87 ± 0.57% vs. 4.50 ± 0.79%, *p* = 0.417; endothelial cells: 7.01 ± 1.16% vs. 6.19 ± 1.01%, *p* = 0.272, Figure 3A). When quantifying the number of PDL1-positive cells in the same experimental approach, there was only a small, albeit significant, difference between DOX-treated and control animals in terms of fibroblasts (2.81 ± 1.20% vs. 9.61 ± 1.11%, *p* = 0.003, Figure 3B) and cardiomyocytes (0.88 ± 0.5% vs. 1.73 ± 0.3%, *p* = 0.031, Figure 3B), mainly due to the rather low expression of PDL1 in these populations. In contrast, immune cells and endothelial cells in the control group showed high expression levels of PDL1, which was lower in DOX-treated animals in both cell types (immune cells: 41.83 ± 10.42% vs. 74.06 ± 6.59%, *p* = 0.003; endothelial cells: 72.83 ± 4.38% vs. 84.37 ± 2.36%, *p* = 0.003, Figure 3B). The number of PD1-positive cells in both groups did not change significantly for immune cells, fibroblasts and cardiomyocytes and showed only a small increase for endothelial cells (mean ± SD: 2.52 ± 0.58% vs. 1.05 ± 0.41%, *p* = 0.003, Figure 3B).

### 2.3. DOX Treatment Does Not Increase Overall Tissue CD31 Expression

**Figure 4:**
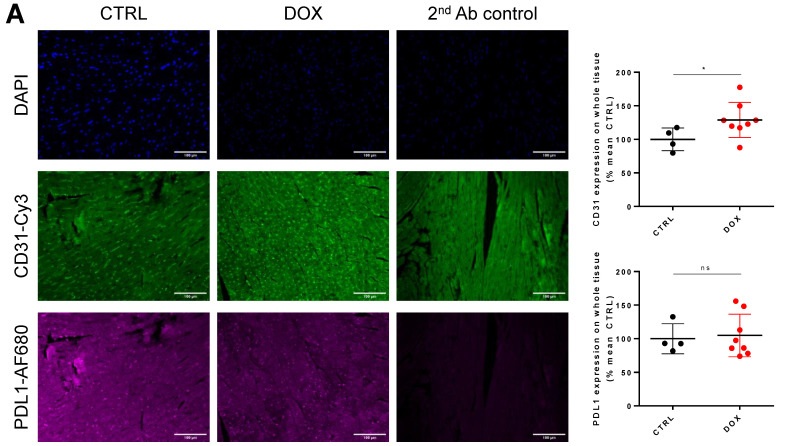

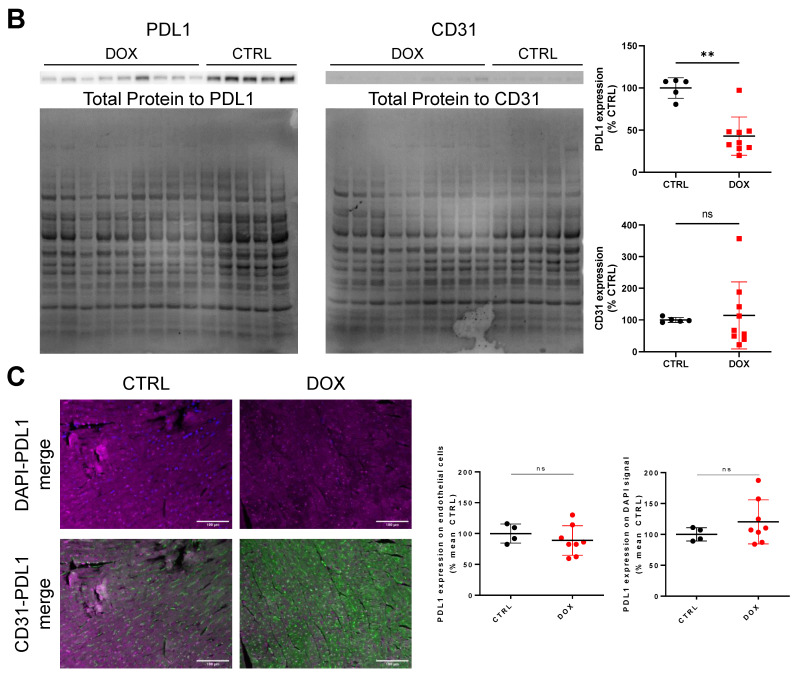
Immuno-histological and Western blot validation of flow cytometry findings. (**A**) Left Exemplary micrographs of DAPI, CD31 and PDL1 expression (with secondary Ab control) in cardiac tissue from control (CTRL) and Doxorubicin (DOX) treated animals. Right Quantified CD31 and PDL1 signal intensity over whole tissue normalized to CTRL group (n = 4–8, Mann–Whitney test, *: *p* < 0.05). (**B**) Left Western blot micrographs stained for PDL1 and CD31 from whole tissue lysates with corresponding total protein stains. Right Quantified expression of PDL1 and CD31 from micrographs on the left normalized to CTRL group (n = 5–9, Mann–Whitney test, **: *p* < 0.01) (**C**) Left and Middle Exemplary merged micrographs of DAPI-PDL1 and CD31-PDL1 from CTRL and DOX groups. Right Quantified PDL1 signal intensity co-localized with DAPI and CD31 signal (n = 4–8, Mann–Whitney test). Scale bars depict 100 µm. ns: not significant.

Following the promising data obtained with flow cytometry, we aimed to further localize the tissue expression of PDL1. Using immuno-histology, we demonstrated that there was a slight increase in CD31-positive structures in DOX-treated animals (128.99 ± 24.39% vs. 100 ± 14.62%, *p* = 0.049, Figure 4A). When testing CD31 expression in whole tissue lysates using Western blot, we did not find a significant increase in the DOX-treated group, although some individual animals showed higher CD31 expression (114.5 ± 105.7% vs. 100 ± 7.9%, *p* = 0.6993, Figure 4B). Overall tissue PDL1 expression was similar in the DOX-treated and control groups (104.86 ± 29.53% vs. 100 ± 19.31%, *p* > 0.999, Figure 4A), showing a homogenous scatter pattern throughout the tissue without clear localization. For PDL1, Western blot analysis of whole tissue lysates revealed decreased PDL1 expression similar to flow cytometry (43.05 ± 22.7% vs. 100 ± 12.17%, *p* = 0.004, Figure 4B). We also examined PDL1 expression levels either in the nucleus (co-localization with DAPI signal) or in endothelial cells (co-localization with CD31). In both cases, there was no significant difference between both groups, although PDL1 expression in endothelial cells showed a slight downward trend (DAPI-PDL1 mean ± SD: 120.20 ± 3.34% vs. 100 ± 9.26%, n = 4–7, *p* = 0.5172; CD31-PDL1 mean ± SD: 88.83 ± 22.36% vs. 100 ± 13.39%, n = 4–7, *p* = 0.5697, Figure 4C).

## 3. Discussion

In our present study, we found that substantial cell damage is detectable in a high-dose model of DOX treatment after 6 days. This damage is associated with decreased heart function and decreased numbers of cardiomyocytes in the hearts of DOX-treated mice. Furthermore, DOX acts on the PD1/PDL1 pathway by decreasing PDL1 expression in several cardiac cell populations, which is not restricted to a specific region in the myocardium but is diffusely distributed throughout the tissue.

The effects of DOX treatment on the PD1/PDL1 pathway in various cancer types remain elusive. Some reports have found increased expression of PDL1 in cancer cells, while others have reported less PDL1 expression [9,27,28]. There are even fewer sources of DOX affecting the PD1/PDL1 pathway in other tissue cells. Given the recent preclinical and clinical studies, focusing on combination therapies of DOX and PD1-PDL1 inhibitors, especially in triple-negative breast cancer, reliable data for adverse effect risk assessment are urgently needed [26,29,30].

We specifically used a high-dose model of DOX injection to take a first look at PD1 and PDL1 expression on cardiac cells to induce cell damage, as previously shown [31,32]. Decreased heart function in DOX-injected animals, as evidenced by reduced FS and EF, was detectable at day 6 after exposure, similar to other recent studies [33,34]. Acute tissue damage was also apparent by the high amounts of CK and cTnI in the blood of DOX-treated animals compared with the control group. Both factors have been described earlier as markers of DOX-induced cardiac injury [35]. Similar, dramatically reduced levels of circulating blood leucocytes were found in the DOX group [36]. Using flow cytometry, we detected a difference in the relative quantities of fibroblasts and cardiomyocytes, whereas there was no such difference in endothelial and immune cells. The decline in cardiomyocytes is consistent with our findings of cell damage markers CK and cTnI in the blood, which indicate cellular damage to cardiomyocytes, and has been shown extensively by other groups [37,38,39,40]. Of note, in one study, DOX was found to cause endothelial cell death in immune-incompetent mice but not decreased cardiomyocyte numbers, which is reversed in our model, hinting at a substantial involvement of the immune system, possibly due to altered PD1/PDL1 signaling [41]. Therefore, we believe that the established model adequately resembles DOX-induced myocardial injury to further study the effects on the PD1-PDL1 pathway.

Following the model analysis, we checked the number of cells expressing either PD1 or PDL1 distributed in these populations. In the control group animals, similar levels of PD1 and PDL1 were measured in all four cell populations as previously published for the baseline mice [20]. In DOX-treated animals, the number of PD1-expressing endothelial cells changed significantly, although it is debatable whether this is also a biologically relevant change. PD1 expression in previous studies of cancer cell types varied following doxorubicin treatment but was increased in a cardiac injury model [12,42,43]. Therefore, PD1 expression in our model may be mediated by cardiac damage rather than doxorubicin signaling. Of note, no cell population expressed a high amount of PD1, not even immune cells. The sharp decrease in PDL1-positive immune cells and endothelial cells requires further research, as it is of high importance when considering combination therapy with PD1/PDL1 pathway inhibitors, at least for considering possible cardiac side effects. PDL1 expression was already studied following DOX treatment, albeit mostly in cancer cells, with heterogeneous results regarding increased or decreased expression [9,28,44].

A limiting factor in this study is the difference between flow cytometry and Western blot on the one hand and immuno-staining on the other with respect to PDL1 expression. To this point, determining PDL1 expression levels on different cardiac cell populations by Western blotting from sorted populations would be an interesting option for further research. Furthermore, this study shows only the cardiotoxic effects of anthracycline therapy, but not actual worsening by sequential or combined ICI therapy to further substantiate our hypothesis.

To conclude, our study is the first to provide a detailed look at the PD1 and PDL1 expression on cardiac cells following DOX treatment. Whereas PD1 expression was scarcely present on any cell in all animals, PDL1 was significantly less expressed by immune cells and endothelial cells after DOX treatment. Especially, the decreased expression on endothelial cells could lead to an increased immune response when anthracycline therapy is combined with or precedes PD1/PDL1 ICI therapy. However, whether this holds true needs to be tested by such combination therapy in an animal model.

## 4. Materials and Methods

### 4.1. Animals and Ethical Statement

C57BL/6JRJ mice (12 ± 3 weeks of age) were housed in the central animal facility of the University Hospital Essen at a 12 h/12 h day-night-cycle and access to food and water ad libitum before and during the experiment. The experiments were approved beforehand by the ethical committee of the Landesamt für Natur-, Umwelt- und Verbraucherschutz (LANUV).

### 4.2. DOX Treatment Model and Experiment Setup

The cardiac function of mice was measured at baseline (day 0) before the beginning of the experiment. Animals were then randomly assigned to DOX or vehicle treatment group. After baseline cardiac assessment, mice in the DOX treatment group received a single intraperitoneal injection of DOX (20 mg/kg; MedacGmbH, Wedel, Germany) in max 150 µL NaCl (B. Braun Melsungen AG, Melsungen, Germany), whereas mice in the vehicle treatment group received a single injection of 150 µL NaCl only. Following published conversions, this DOX dose equals ~55 mg/m^2^ [45,46]. Mice were weighed and visually investigated every day to check for worsening overall fitness. On day 6 after injection, mice were again measured for cardiac function. Afterward, animals were killed by exsanguination in deep isoflurane (Baxter GmbH, Unterschleißheim, Germany) narcosis after injection of Ketamine/Xylazine (100/20 mg/kg; Hameln Pharma, Hameln, Germany/Bela Pharm, Vechta, Germany). The heart was extracted after perfusing the animal free of blood with PBS without calcium or magnesium (PBS^−Ca/−Mg^; ThermoFisher Scientific, Waltham, MA, USA) including 20 IE/mL of heparin (Leo Pharma GmbH, Neu-Isenburg, Germany). From the extracted heart, a single midventricular slice (~2 mm thickness) was obtained for histology and fixated in 4% PFA at RT overnight, while the rest of the heart was used for flow cytometry.

### 4.3. Cardiac Function and Blood Analysis

Mouse echocardiography was conducted using a Visualsonics Vevo 3100 Imaging system (FUJIFILM Visualsonics, Toronto, ON, Canada). After initial anesthesia with 4 Vol.-% isoflurane in 1 L/min O_2_, mice were placed on a heated plate and anesthesia was maintained with 1.5 Vol.-% isoflurane. A rectal probe was introduced, and heart rate, respiratory rate and body temperature were monitored continuously. After hair removal, images of long and short parasternal axis were acquired in M-mode and B-mode. Measurements were analyzed offline by a blinded investigator using Visualsonics VevoLab 3.2.6 software (FUJIFILM Visualsonics, Toronto, ON, Canada). Fractional shortening was determined by comparison of midventricular short-axis enddiastolic and endsystolic volumes. The ejection fraction was calculated using LV tracing on the parasternal short axis [47,48]. CK and cTnI levels were measured from serum samples using high-sensitive ELISA assays (both Siemens, Erlangen, Germany).

### 4.4. Flow Cytometry

Hearts were mechanically minced and incubated in an enzyme solution comprised of 450 U/mL collagenase I (Sigma Aldrich, St. Louis, MO, USA), 125 U/mL collagenase XI (Sigma Aldrich, St. Louis, MO, USA), 60 U/mL hyaluronidase (Sigma Aldrich, St. Louis, MO, USA), 20 mM HEPES (Sigma Aldrich, St. Louis, MO, USA), 60 U/mL DNase (Sigma Aldrich, St. Louis, MO, USA) in PBS with Calcium and Magnesium (PBS^Ca/Mg^_,_ ThermoFisher Scientific, Waltham, MA, USA) for 40 min in a ThermoMixer C (Eppendorf, Hamburg, Germany) at 37 °C and 750 r.p.m. After enzymatic digestion, the solution was filtered through a 40 μm filter and flow-through was centrifuged for 5 min at 4 °C and 400× *g*. The supernatant was discarded and the cell pellet dissolved in 1.5 mL PBS^−Ca/−Mg^; 200 μL of this solution per sample was used for staining, while a mixture of all solutions was used for fluorescence minus one (FMO) control. Blocking of FC-receptors was conducted by adding 2 μL of TruStain fcX (BioLegend, San Diego, CA, USA) and incubating for 20 min on ice in the dark. Samples were washed by adding 100 μL PBS^−Ca/−Mg^, centrifuging for 5 min at 4 °C and 400× *g*, discarding the supernatant, and dissolving the cell pellet in 50 μL PBS^−Ca/−Mg^. Samples were stained by adding 50 μL PBS^−Ca/−Mg^ containing antibodies CD45-AF700 (BioLegend, San Diego, CA, USA), CD31-BV421 (BioLegend, San Diego, CA, USA), CD140a-BV605 (BioLegend, San Diego, CA, USA), PD1-APC (BioLegend, San Diego, CA, USA) and PDL1-PE (BioLegend, San Diego, CA, USA), all at a dilution of 1:200 except for PDL1-PE which was at 1:400. Incubation was conducted for 30 min at RT in the dark. After antibody incubation, samples were washed as before, the supernatant was discarded and the cell pellet was dissolved in 300 μL FACS-Buffer, comprised of 1% (*v*/*v*) fetal bovine serum (PAN Biotech, Aidenbach, Germany) and 0.5% bovine serum albumin (Roth, Karlsruhe, Germany) in PBS^−Ca/−Mg^. Data were acquired on a BD FACS Aria III (BD Biosciences) and analysis was performed using FlowJo 10.8.1 software (BD Life Sciences, East Rutherford, NJ, USA).

### 4.5. Immuno-Histology

After overnight fixation, obtained midventricular slices were dehydrated in an ascending ethanol series (50%, 70%, 100% *v*/*v* in ddH_2_O, Roth, Karlsruhe, Germany) at RT for 1 h each, followed by two changes of isopropyl alcohol (Roth, Karlsruhe, Germany) and one change of xylene (Roth, Karlsruhe, Germany) at RT for 1 h each, before overnight incubation in one additional change of xylene (Roth, Karlsruhe, Germany). Afterward, samples were embedded in paraffin and 5 µm histological slices were obtained using a microtome. Three histological slices per sample were used for staining and an additional three slices from one sample were used as a secondary antibody control. Samples were deparaffinized in an oven at 60 °C for 20 min and rehydrated in two changes of xylene (Roth, Karlsruhe, Germany), and a descending ethanol series (100%, 100%, 96%, 96%, 70%, 0% *v*/*v* in ddH_2_O, Roth, Karlsruhe, Germany) for 3 min each, followed by an antigen retrieval procedure in citrate buffer (Sigma-Aldrich, St. Louis, MO, USA) at 95 °C for 30 min. Samples were allowed to cool to RT for 20 min in the citrate buffer. Samples were washed in three changes of tap water, two changes of ddH_2_O and one change of tris-buffered saline, containing 10 mM TRIS (Roth, Karlsruhe, Germany) and 100 mM NaCl (Roth, Karlsruhe, Germany) in ddH_2_O, with 0.1% (*v*/*v*) Tween-20 (Roth, Karlsruhe, Germany; TBS-T) for 5 min each. Blocking was conducted in TBS-T + 5% normal goat serum (Invitrogen, Waltham, MA, USA; NGS) at RT for 1 h. Samples were briefly rinsed with TBS-T and then incubated with primary antibodies for CD31 (rat, 1:20, Dianova, Hamburg, Germany) and PDL1 (rabbit, 1:50, Invitrogen, Waltham, MA, USA) in TBS-T + 5% NGS at 4 °C overnight. The secondary antibody control was incubated in TBS-T + 5% NGS only. Samples were again briefly rinsed with TBS-T and then washed thrice in TBS-T for 5 min each. Staining with secondary antibodies for rat (goat, 1:200, Cy3, Invitrogen, Waltham, MA, USA) and rabbit (goat, 1:200, AlexaFluor 680, Invitrogen, Waltham, MA, USA) was conducted in TBS-T + 5% NGS at RT for 1 h. Afterward, samples were rinsed with TBS-T and then washed thrice in TBS-T for 5 min each. Nuclear staining was conducted with DAPI (Invitrogen, Waltham, MA, USA) 1:5000 in TBS-T at RT for 5 min, followed by washing samples in TBS-T thrice for 5 min each. Samples were preserved using Prolong Gold Antifade (Invitrogen, Waltham, MA, USA) before and after imaging. Two images from brightfield (BF), DAPI, CD31-Cy3 and PDL1-AlexaFluor 680 of each slice (three slices per animal, resulting in six images per animal) were obtained using an EVOS microscope (ThermoFisher Scientific, Waltham, MA, USA) using the transmitted light setting and the DAPI, RFP and Cy5 EVOS light cubes at 20× magnification. Tissue outlines were traced and fluorescence intensity of CD31-Cy3 and PDL1-AlexaFluor 680 were measured using ImageJ 1.53 software (NIH, New York City, NY, USA) [49]. Similarly, DAPI and CD31-Cy3^high^ signals were traced and PDL1-AlexaFluor 680 intensity was measured in these areas using ImageJ software [49]. Signal intensities were normalized to the mean of CTRL group values.

### 4.6. Western Blot

Hearts were mechanically minced and incubated in RIPA buffer comprised of 1% NP40 (Sigma, St. Louis, MO, USA), 150 mM NaCl (Roth, Karlsruhe, Germany), 0,5 mM EDTA (Roth, Karlsruhe, Germany), 50 mM TRIS-HCL (Roth, Karlsruhe, Germany) and 1% protease/phosphatase inhibitor (Thermo Scientific, Waltham, MA, USA) in PBS^−Ca−Mg^ pH = 7.4 for 1 h at 4 °C on a rotor at 20 rpm. Afterward, suspensions were centrifuged at 20,000× *g* for 15 min at 4 °C and supernatants were stored at −80 °C until further analysis. Protein concentration was determined using a Bradford Assay (Bio-Rad, Hercules, CA, USA) and 20 µg protein per sample were transferred to a 4–12% Bis-Tris-Glycine gel (Invitrogen, Waltham, MA, USA). SDS-PAGE was run at a constant 120 V for 80 min. Proteins were dry transferred from the gel to a nitrocellulose membrane using the iBlot2 system (Invitrogen, Waltham, MA, USA) at P0 (20 V for 1 min, 23 V for 4 min, 25 V for 2 min). For the total protein stain, membranes were fixed in 7% acetic acid (Roth, Karlsruhe, Germany), and 10% methanol (Sigma, St. Louis, MO, USA) in dH_2_O for 15 min at RT. Following four washing steps at 5 min each in dH_2_O, proteins were stained using Novex SYPRO Ruby Membrane Stain (Invitrogen, Waltham, MA, USA) for 15 min at RT. Membranes were then washed four times for 1 min each in dH_2_O and imaged using an Amersham680 imager (GE LifeSciences, Washington, DC, USA). Membranes were then blocked with 3% bovine serum albumin (Roth, Karlsruhe, Germany) in tris-buffered saline comprised of 10 mM TRIS (Roth, Karlsruhe, Germany), 100 mM NaCl (Roth, Karlsruhe, Germany) and 2% Tween-20 (Roth, Karlsruhe, Germany) in dH_2_O at pH 7.5 (TBS-T buffer) for 1 h at RT. Staining was conducted in a new change of the same buffer with 3% serum overnight at 4 °C with antibodies for PDL1 (rabbit, 1:1000, Invitrogen, Waltham, MA, USA) and CD31 (rat, 1:5000, Dianova, Hamburg, Germany). Membranes were washed three times in TBS-T buffer at RT and then secondary antibodies conjugated with horse radish peroxidase were added in TBS-T buffer plus 3% bovine serum albumin (Roth, Karlsruhe, Germany) for 1 h at RT (anti-rabbit, 1:5000, Invitrogen, Waltham, MA, USA; anti-rat, 1:5000, Abcam, Cambridge, UK). Afterward, membranes were washed three times in TBS-T buffer and imaged using an Amersham680 imager (GE LifeSciences, Washington, DC, USA) with Pierce ECL Pico Western Blotting Substrate (Thermo Scientific, Waltham, MA, USA). Images were analyzed using ImageJ 1.53 (NIH, New York City, NY, USA) [49]. Intensity values obtained from antibody stainings were first normalized to values from total protein stains and then normalized to mean values from the control group.

### 4.7. Statistics

Statistical analysis was conducted with Graphpad Prism 6 (Graphpad Software, Boston, MA, USA). All graphs show every biological individual analyzed, in addition to the mean and standard deviation of the respective group. Values were tested for Gaussian distribution and tests were chosen accordingly (Mann–Whitney or Student’s *t*-test for single comparison, Kruskal–Wallis with Dunn’s correction or ANOVA with Bonferroni correction for multiple comparisons) as stated in the respective figure legend. A *p*-value <0.05 was considered statistically significant and significance levels were marked with asterisks (<0.05 *, <0.01 **, <0.001 ***).

## Data Availability

The raw data for the findings provided in the publication are available from the corresponding author upon reasonable request.
